# Prevalence of *Streptococcus mutans* harboring the *cnm* gene encoding cell surface protein Cnm in Japanese children

**DOI:** 10.1038/s41598-025-11478-w

**Published:** 2025-07-25

**Authors:** Yuto Suehiro, Makoto Okuda, Masatoshi Otsugu, Marin Ochiai, Misato Takagi, Fumikazu Tojo, Yusuke Mikasa, Shuhei Naka, Michiyo Matsumoto-Nakano, Jinthana Lapirattanakul, Rena Okawa, Ryota Nomura, Kazuhiko Nakano

**Affiliations:** 1 Department of Pediatric Dentistry, Graduate School of Dentistry, The University of Osaka, 1-8 Yamada-Oka, Suita, Osaka 565-0871 Japan; 2 Department of Pediatric Dentistry, Graduate School of Medicine, Dentistry and Pharmaceutical Sciences, Okayama University, Okayama, Japan; 3 Department of Oral Microbiology, Faculty of Dentistry, Mahidol University, Bangkok, Thailand; 4 Department of Pediatric Dentistry, Graduate School of Biomedical and Health Sciences, Hiroshima University, Hiroshima, Japan

**Keywords:** *Streptococcus mutans*, Collagen-binding protein, Cnm, Prevalence, Dental caries, Japanese population, Microbiology, Bacteriology

## Abstract

Dental caries is a highly prevalent infectious disease primarily caused by the pathogenic bacterium *Streptococcus mutans*, which has also been associated with systemic disease. A 120-kDa collagen-binding protein (Cnm) produced by *S. mutans* contributes to cardiovascular disease pathogenicity. Few studies have addressed the current prevalence of *S. mutans* and the *cnm* gene in Japanese children or examined caries pathology in relation to *cnm* presence. Here, we investigated the prevalence of *S. mutans* and the distribution of *cnm*-positive *S. mutans* among 490 children who visited two university hospitals in Japan. The caries experience index (dmft/DMFT) was calculated, and the collagen-binding ability of *cnm*-positive *S. mutans* strains was assessed. *S. mutans* was isolated from the oral cavities of 158 patients (36.8%); 10.1% (16/158) harbored *cnm*-positive *S. mutans*. When caries experience indices were compared across dentitions, patients harboring *cnm*-positive strains had significantly higher dmft/DMFT scores than those with *cnm*-negative strains (*P* < 0.05). Additionally, a positive correlation was observed between the collagen-binding capacity of *cnm*-positive *S. mutans* and the dmft/DMFT score (r = 0.601, *P* < 0.05). These findings suggest that *cnm* contributes to caries progression through collagen-mediated adherence to tooth surfaces. The presence of *cnm*-positive *S. mutans* may represent a risk factor for increased caries susceptibility in children.

## Introduction

Dental caries is a common oral disease worldwide^1^. Its prevalence has substantially declined (by approximately 20–60%, depending on region) among young children, adolescents, and adults over the past 30–40 years^2^. Contributing factors include improved oral hygiene resulting from enhanced public awareness of oral health, better dietary habits regarding sucrose consumption, greater use of fluoride—particularly through widespread toothpaste use—and an increase in the number of individuals receiving regular dental check-ups^2^.

*Streptococcus mutans*, a bacterium implicated in dental caries, has been associated with systemic diseases such as infective endocarditis (IE)^3^. *Streptococcus mutans* is serologically classified into four serotypes—*c*, *e*, *f*, and *k*—based on structural differences in the polysaccharide antigen composed of glucose and rhamnose polymers present on the bacterial surface^4,5^. In the oral cavity, serotype *c* is most frequently observed (approximately 75%), followed by serotype *e* (approximately 20%), whereas serotypes *f* and *k* each occur at rates below 5%^5–7^. Cnm, a 120-kDa collagen-binding protein encoded by the *cnm* gene, is expressed on the surface of *S. mutans* strains isolated from the oral cavity at a frequency of approximately 10–20%^8,9^. *Streptococcus mutans* strains harboring the *cnm* gene can bind to collagen within the dentin and vascular wall via Cnm, a process closely linked to the pathogenicity of caries and IE^10,11^.

Recently, in Japan, public interest in oral hygiene has increased, particularly for the prevention of both oral and systemic diseases^12,13^. Although the prevalence of dental caries has declined, the current rate of *S. mutans* carriage in children remains unclear. Additionally, as previously mentioned, *cnm*-positive strains exhibit collagen-binding capacity and are therefore presumed to carry a higher caries risk than *cnm*-negative strains. However, limited data are available regarding the recent prevalence of *cnm*-positive strains. Thus, the present study investigated the current prevalence of *S. mutans* and the distribution of *cnm*-positive *S. mutans* in the oral cavities of Japanese children across a broad age range (1–19 years), then analyzed the associations of these factors with caries experience (decayed, missing, and filled primary teeth [dmft]/decayed, missing, and filled permanent teeth [DMFT]). Additionally, we evaluated the collagen-binding ability of the detected *cnm*-positive *S. mutans* strains and explored the correlation with dmft/DMFT.

## Results

### Study design

**Fig. 1:**
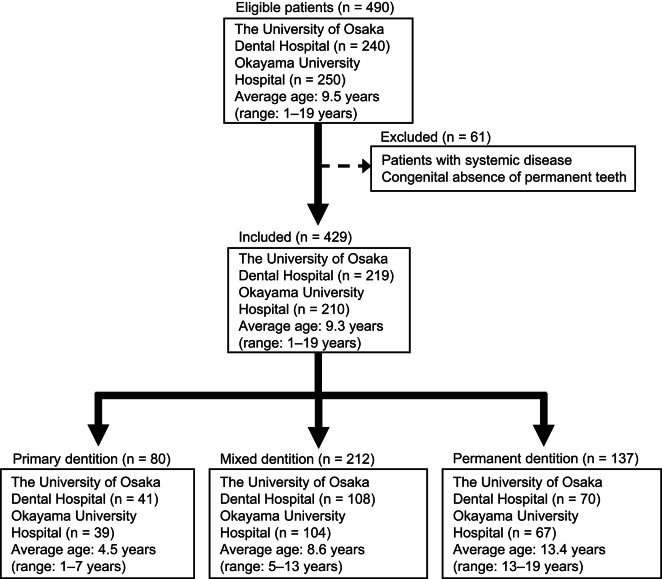
Flowchart of patient selection.

The flowchart of patient selection is shown in Fig. 1. A total of 490 patients who visited the Pediatric Dentistry Clinic at The University of Osaka Dental Hospital or Okayama University Hospital for treatment or check-ups were initially enrolled. Among these, 61 patients were excluded based on the presence of systemic disease. The final study cohort consisted of 429 patients (Fig. 1). These patients were subsequently classified into three groups according to dentition: deciduous (n = 80), mixed (n = 212), and permanent (n = 137). The dmft/DMFT indices were calculated for each group (Fig. 1).

### Prevalence of *S. mutans *and *cnm*-positive *S. mutans*

Among all patients, *S. mutans* was isolated from the oral cavities of 158 individuals (36.8%). Specifically, *S. mutans* was detected in 26 patients (32.5%) with primary dentition, 81 patients (38.2%) with mixed dentition, and 51 patients (37.2%) with permanent dentition. Among those in whom *S. mutans* was detected, 16 individuals (10.1%) harbored *cnm*-positive *S. mutans*, comprising four patients (15.4%) in the primary dentition group, nine (11.1%) in the mixed dentition group, and three (5.9%) in the permanent dentition group (Table 1). Serotyping of the 16 *cnm*-positive *S. mutans* strains revealed that 10 strains (62.5%) were serotype *c*, two strains (12.5%) were serotype *e*, three strains (18.8%) were serotype *f*, and one strain (6.3%) was serotype *k* (Table 2).

**Table 1:** *S. mutans* and *cnm* positivity rates by dentition stage.

Dentition stage	Number of patients	*S. mutans*-positive	*cnm*-positive
Primary (1–7 years)	80 (18.6%)^#^	26 (32.5%)^##^	4 (15.4%)^###^
Mixed (5–13 years)	212 (49.4%)^#^	81 (38.2%)^##^	9 (11.1%)^###^
Permanent (10–19 years)	137 (31.9%)^#^	51 (37.2%)^##^	3 (5.9%)^###^
Total	429 (100%)	158 (36.8%)^#^	16 (10.1%)^###^

^#^Percentage of all patients, ^##^percentage in each dentition stage, ^###^ percentage of *S. mutans*-positive individuals in each dentition stage.

**Table 2:** Characteristics of *cnm*-positive individuals.

Strain	Serotype	Age	dmft	DMFT	Collagen-binding rate
OS1	*c*	6	12	0	123.1
OS52	*c*	9	10	0	119.3
OS56	*e*	10	5	1	114.4
OS60	*c*	10	2	2	73.5
OS67	*e*	4	11	0	172.1
OS77	*c*	12	4	4	91.1
OS125	*c*	11	0	9	92.4
OS131	*c*	3	12	0	141.7
OS150	*f*	7	8	0	92.0
OS180	*c*	6	9	0	100.3
OS186	*c*	13	2	8	120.2
OS219	*c*	11	0	4	75.7
OK38	*f*	15	0	11	95.0
OK86	*f*	5	16	0	166.4
OK120	*c*	7	6	0	158.4
OK212	*k*	6	14	0	130.2

dmft, decayed, missing, and filled primary teeth; DMFT, decayed, missing, and filled permanent teeth.

### Comparison of the number of teeth affected by dental caries in each dentition

**Fig. 2:**
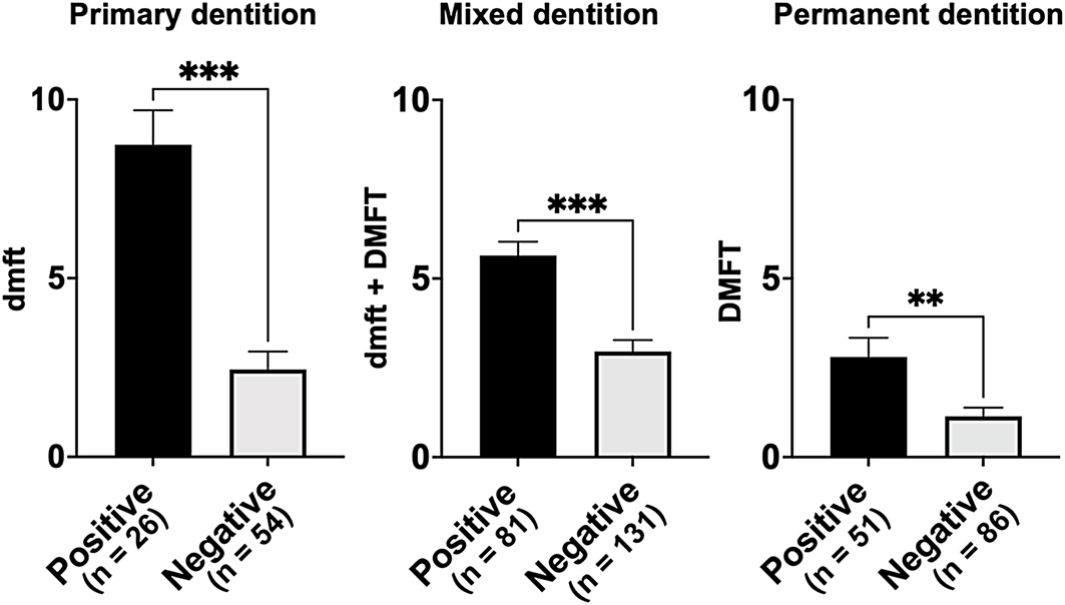
Comparison of the number of teeth affected by dental caries in each dentition between patients with and without *S. mutans* Significant differences were observed in all dentitions using analysis of variance with Bonferroni correction (***P < 0.001, **P < 0.01).

**Fig. 3:**
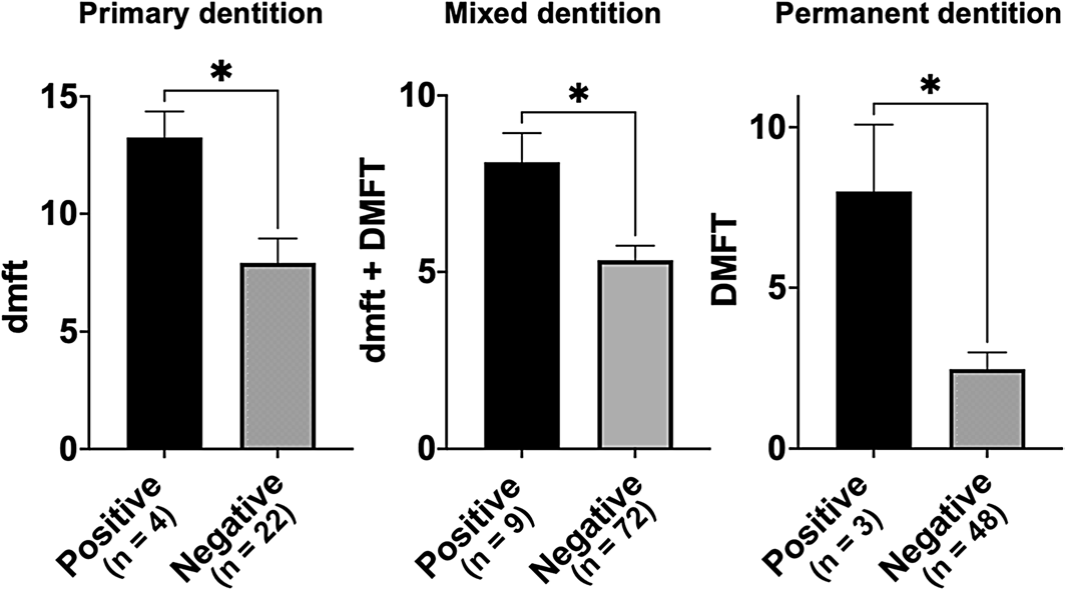
Comparison of the number of teeth affected by dental caries in each dentition among *S. mutans*-positive patients with and without *cnm* Significant differences were observed in all dentitions using analysis of variance with Bonferroni correction (*P < 0.05).

When the caries experience index was compared based on the presence or absence of *S. mutans*, *S. mutans*-positive individuals (primary: 8.7 ± 0.6, mixed: 5.6 ± 0.2, permanent: 2.8 ± 0.3) exhibited significantly higher indices than *S. mutans*-negative individuals (primary: 2.4 ± 0.4, mixed: 3.0 ± 0.3, permanent: 1.1 ± 0.2) across all dentitions (*P* < 0.001 in primary and mixed dentitions; *P* < 0.01 in permanent dentition) (Fig. 2). Similarly, when comparisons were made according to the presence of *cnm*, individuals with *cnm*-positive *S. mutans* showed significantly higher caries experience indices (primary: 13.3 ± 0.3, mixed: 8.1 ± 0.2, permanent: 8.0 ± 0.3) than those without *cnm*-positive strains (primary: 7.9 ± 0.5, mixed: 5.3 ± 0.2, permanent: 2.5 ± 0.3) in all dentitions (*P* < 0.05) (Fig. 3).

### Correlation between collagen-binding ability of *cnm*-positive strains and dmft/DMFT

**Fig. 4:**
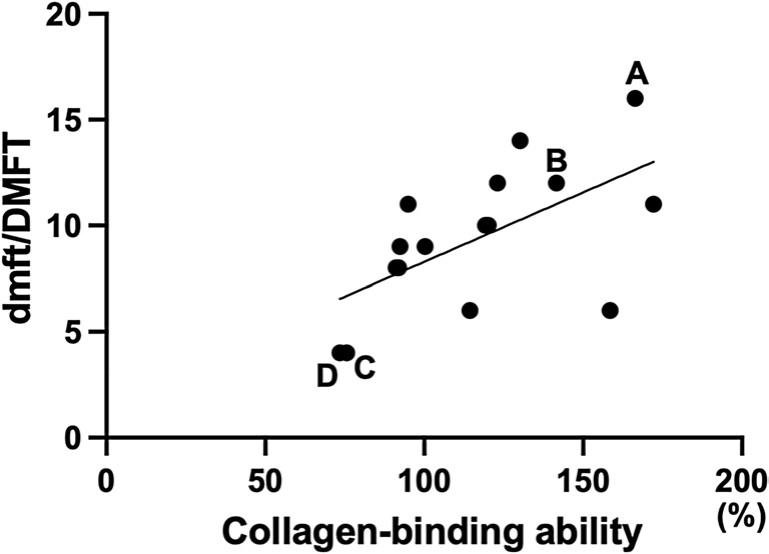
Correlation between the collagen-binding ability of *cnm*-positive *S. mutans* and the dmft/DMFT scores A positive correlation was observed between the collagen-binding rate and the dmft/DMFT score (r = 0.601, *P* < 0.05). Representative strains with the highest OK86 (**A**) and OS131 (**B**) and lowest OS60 (**C**) and OS219 (**D**) dmft/DMFT scores and collagen-binding abilities are shown.

The collagen-binding rates of *cnm*-positive *S. mutans* strains varied, ranging from 73.1 to 171.8% (Table 2). A positive correlation was observed between the collagen-binding rate and the dmft/DMFT index (r = 0.601, *P* < 0.05) (Fig. 4).

### Morphological evaluation of *cnm*-positive *S. mutans*

**Fig. 5:**
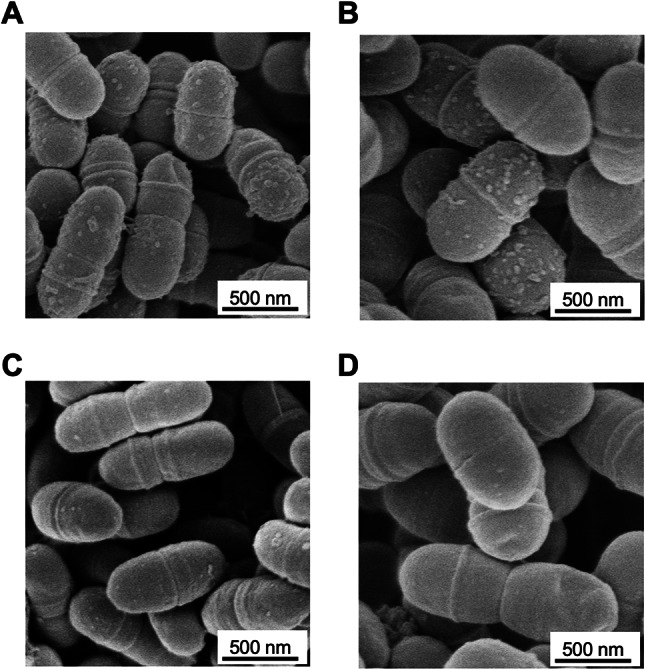
Representative scanning electron microscopy images of *cnm*-positive *S. mutans* Strains OK86 (**A**) and OS131 (**B**) exhibited numerous protrusions on the cell surface, whereas strains OS60 (**C**) and OS219 (**D**) showed fewer surface protrusions. Scale bar = 500 nm.

Among the *cnm*-positive *S. mutans* strains, two strains (OK86 and OS131) with high collagen-binding ability and high dmft/DMFT scores in their respective hosts were selected. For comparison, two strains (OS60 and OS219) with low collagen-binding ability and low dmft/DMFT scores were also examined. Scanning electron microscopy (SEM) revealed numerous protrusions on the cell surfaces of OK86 and OS131 (Fig. 5A, B), whereas OS60 and OS219 exhibited fewer surface protrusions (Fig. 5C, D).

## Discussion

*S. mutans* is a major causative organism of dental caries^14^. The presence of the Cnm protein, encoded by the *cnm* gene and expressed on the surface of *S. mutans*, has been identified as a factor contributing to bacterial adhesion to dentin^10^*.* The prevalence of dental caries has been declining over time^15,16^. In this study, we investigated the prevalence of *S. mutans* harboring *cnm* and the number of teeth affected by caries in a recent cohort of Japanese children; we also analyzed the relationship between Cnm and caries incidence. Our results allowed us to clarify the current *cnm*-positivity rate among Japanese children and demonstrate an association between Cnm and caries.

Samples were collected from children under 20 years of age in two distinct regions of Japan (urban: Osaka; rural: Okayama) to investigate potential regional differences. In total, 490 children were enrolled, with approximately equal numbers from both locations. Patients with systemic disease were excluded, given that *cnm*-positive *S. mutans* has been detected more frequently in individuals with systemic disease^17–19^, and medications used in such cases may influence the oral microbial flora. After exclusion, 429 patients remained and were categorized into three groups according to dentition stage: deciduous (1–7 years), mixed (5–13 years), and permanent (13–19 years). Caries experience indices (dmft, dmft + DMFT, and DMFT) were calculated for each group.

The prevalence of *S. mutans* in this study was 36.8%, considerably lower than the 70–80% reported in previous studies^20^. Two factors may explain this discrepancy. First, the overall incidence of dental caries has been decreasing, as previously reported^16^. Second, bacterial sampling in this study was performed using oral swabs, which may yield lower detection rates relative to saliva samples^20^. Saliva samples are thought to reflect bacterial populations throughout the oral cavity^21^. Therefore, differences in microbial yield and composition between swab and saliva samples may have influenced the detection and strain representation of *S. mutans* in this study. No significant differences in *S. mutans* prevalence were observed among the different dentition groups. Although previous research has suggested that the prevalence of *S. mutans* increases with age^22^, the present findings indicate that such a trend may not be evident among children.

The proportion of *cnm*-positive *S. mutans* carriers among those positive for *S. mutans* was 10.1%, a prevalence consistent with previous reports^8^. This suggests that the prevalence of *cnm*-positive *S. mutans* has remained relatively stable over time. Notably, in the present study, the prevalence of *cnm*-positive *S. mutans* decreased with age. Although earlier studies have reported a higher prevalence of *cnm*-positive *S. mutans* in adults relative to children^8^, no studies (to our knowledge) have examined this trend across different age groups within a pediatric population. Based on the present findings, the prevalence of *cnm*-positive *S. mutans* may temporarily decrease with the loss of primary teeth and subsequently increase with age.

Concerning the dmft/DMFT index, *S. mutans*-positive individuals exhibited significantly higher scores across all dentitions compared with *S. mutans*-negative individuals. Similarly, individuals harboring *cnm*-positive *S. mutans* demonstrated significantly higher dmft/DMFT scores than those without *cnm*-positive strains in all dentition groups. Previous studies have indicated that *cnm*-positive *S. mutans* may contribute to caries progression by adhering to exposed collagen in dentin^10^, and the present findings support this hypothesis. It is worth noting that the dmft/DMFT index reflects lifetime caries experience but is not able to show the current activity of carious lesions. Therefore, although the results of this study have indicated an association between lifetime caries experience and the current presence of *S. mutans* and *cnm*, additional evaluations, such as International Caries Detection and Assessment System (ICDAS) and visual-tactile assessment of lesions, may be informative regarding caries progression and activity.

In both comparisons—based on the presence or absence of *S. mutans* and the presence or absence of *cnm*-positive *S. mutans*—dmft/DMFT scores were consistently higher in the younger age groups and lower in older age groups. This pattern may be attributed to the structural characteristics of primary teeth, which possess thinner enamel and dentin layers than permanent teeth, rendering them more susceptible to caries^23^. Additionally, older patients may have benefited from longer histories of dental visits and more consistent oral care, contributing to reduced experience of caries.

Furthermore, previous studies have demonstrated that the collagen-binding ability of *S. mutans* differs according to the presence or absence of Cnm^8^. The present study also revealed that this ability varies substantially among strains, even within *cnm*-positive *S. mutans*. A positive correlation was observed between the collagen-binding ability of *cnm*-positive *S. mutans* and the dmft/DMFT index, suggesting that carriers of strains with higher collagen-binding capacity tend to have greater experience of caries. Electron microscopy of *cnm*-positive *S. mutans* revealed the presence of surface protrusions, consistent with previous reports^24^. Notably, strains with greater collagen-binding capacity displayed more prominent surface protrusions. This finding suggests an association between collagen-binding ability and cell surface structure. These results indicate that *cnm* may contribute to caries progression through collagen-mediated adhesion to tooth structures, and the presence of *cnm*-positive strains represents a factor that increases caries risk. In addition to identifying the presence of *S. mutans*, approaches that evaluate the collagen-binding ability of the detected strains may enhance assessment of individual caries risk.

The present study has several limitations. First, although it would have been preferable to collect saliva samples from all patients and analyze them along with swab samples, the inclusion of young children made saliva collection impractical. Therefore, swab samples were used. Future studies should consider collecting both swab and saliva samples from children of an age at which saliva sampling is feasible. Second, this study exclusively focused on children, which may explain discrepancies with findings from previous studies. Future research will include adults to evaluate the current prevalence of *cnm*-positive *S. mutans* in the adult population. Third, although this study solely focused on *S. mutans*, analyses of other oral bacterial species might have enabled a more detailed examination of the relationship between microbial composition and caries. Finally, a more comprehensive assessment could have been achieved by incorporating data concerning variables beyond dmft/DMFT, such as ICDAS, dietary habits, oral hygiene practices, and infant feeding history. Nevertheless, this study clarified the current prevalence of *S. mutans* and *cnm* in Japanese children; it also provided evidence supporting the association between collagen-binding ability and dmft/DMFT. Future studies will compare these findings with recent data from adult populations. Subsequent investigations will also include molecular epidemiological analyses to facilitate a more detailed understanding of the mechanisms involved.

## Methods

### Ethics statement

This study was conducted in full accordance with the Declaration of Helsinki. The study protocol was approved by the Ethics Committee of Graduate School of Dentistry, The University of Osaka (approval no. R1-E51) and the Ethics Committee of Okayama University Graduate School of Medicine, Dentistry and Pharmaceutical Sciences (approval no. 2012-007). All parents were informed of the study details through written materials and verbal explanations. Written informed consent was obtained from all parents prior to the enrollment of their children in the study. Furthermore, verbal assent was obtained from all participating children, and written informed assent was obtained from those aged > 6 years.

### Patients and sample collection

In total, 490 children (1–4 years: 40 patients, 5 years: 41 patients, 6 years: 42 patients, 7 years: 43 patients, 8 years: 41 patients, 9 years: 41 patients, 10 years: 40 patients, 11 years: 42 patients, 12 years: 40 patients, 13 years: 40 patients, 14 years: 40 patients, 15–19 years: 40 patients) who attended the Pediatric Dentistry Clinic at the University of Osaka Dental Hospital, Suita, Osaka, Japan (240 patients), or Okayama University Hospital, Okayama, Okayama, Japan (250 patients) between 2018 and 2023 were initially enrolled in the study (Fig. 1). Among them, 429 patients (1–4 years: 37 patients, 5 years: 39 patients, 6 years: 38 patients, 7 years: 39 patients, 8 years: 35 patients, 9 years: 37 patients, 10 years: 35 patients, 11 years: 36 patients, 12 years: 37 patients, 13 years: 34 patients, 14 years: 30 patients, 15–19 years: 32 patients) without systemic diseases were selected for inclusion. *S. mutans* was isolated from dental plaque specimens collected from all fully erupted teeth and erupting teeth using sterile swabs (Eiken Chemical Co. Ltd, Tochigi, Japan), in accordance with a conventional protocol^25^.

### Bacterial isolation

*S. mutans* was isolated from swab samples and quantified as previously described^26^. The swabs were subjected to tenfold and 100-fold serial dilutions in saline solution and plated onto mitis salivarius (MS) agar (Difco Laboratories, Detroit, MI, USA) supplemented with bacitracin (0.2 U/ml; Sigma-Aldrich Co., St. Louis, MO, USA) and 15% (wt/vol) sucrose (MSB agar plates). Plates were incubated at 37 °C for 2 days under an atmosphere consisting of 95% N_2_ and 5% CO_2_. Colonies resembling *S. mutans* were identified on MSB agar based on their characteristic rough morphology^27^.

### DNA extraction

Genomic DNA was extracted from each strain as previously described^28^. Briefly, bacterial cells cultured in brain–heart infusion broth were harvested and incubated with 62.5 μl of lysozyme chloride from chicken egg white (2.0 mg/ml; Sigma-Aldrich Co.) and 0.25 μl of lysozyme hydrochloride from chicken egg white (10 mg/ml; Fujifilm Wako Pure Chemical Industries, Osaka, Japan) for 90 min at 37 °C. For DNA extraction, samples were incubated in 600 μl of Cell Lysis Solution (Qiagen, Düsseldorf, Germany) at 80 °C for 5 min, followed by the addition of 3 μl of RNase A (10 mg/ml; Qiagen) and incubation at 37 °C for 30 min. Subsequently, 200 μl of Protein Precipitation Solution (Qiagen) was added, vortexed vigorously for 20 s, and centrifuged at 10,000 × g for 3 min. The supernatant was transferred to a new tube, combined with 600 μl of isopropanol (Fujifilm Wako Pure Chemical Industries), and centrifuged. The resulting precipitate was washed with 70% ethanol (Fujifilm Wako Pure Chemical Industries), centrifuged, and resuspended in 100 μl of DNA Hydration Solution (Qiagen), then stored as the DNA extract.

### Confirmation of *S. mutan*s and *cnm*

Confirmation of *S. mutans* and detection of the collagen-binding gene (*cnm*) were performed by polymerase chain reaction (PCR) using TaKaRa Ex Taq polymerase (Takara Bio, Shiga, Japan). *S. mutans*-specific primers (forward: 5′-GGC ACC ACA ACA TTG GGA AGC TCA GTT-3′; reverse: 5′-GGA ATG GCC GCT AAG TCA ACA GGA T-3′)^29^ and *cnm*-specific primers (forward: 5′-GAC AAA GAA ATG AAA GAT GT-3′; reverse: 5′-GCA AAG ACT CTT GTC CCT GC-3′)^8^ were used, along with template DNA and 1.5 mM MgCl_2_, following the manufacturer’s protocol. A list of primers used in this study is provided in Table 3. Amplification was carried out under the following conditions: for *S. mutans*, 30 cycles of denaturation at 98 °C for 10 s, followed by primer annealing and extension at 70 °C for 1 min; for *cnm*, initial denaturation at 95 °C for 4 min, followed by 30 cycles of 95 °C for 30 s, 60 °C for 30 s, and 72 °C for 2 min, with a final extension at 72 °C for 7 min. PCR products were subjected to electrophoresis on 1.5% or 0.7% agarose gels in Tris–acetate-EDTA buffer. Gels were stained with 0.5 μg/ml ethidium bromide and visualized under ultraviolet illumination. Strains MT8148 (*cnm*-negative) and TW295 (*cnm*-positive) were used as controls in the PCR assays. Samples exhibiting positive amplification for *cnm* were classified as *cnm*-positive.

**Table 3:** Primers used in this study.

Purpose of detection	Sequence (5′–3′)	Size (bp)	References
*S. mutans*	5′-GGCACCACAACATTGGGAAGCTCAGTT-3′	433	28,29
	5′-GGAATGGCCGCTAAGTCAACAGGAT-3′		
*cnm*	5′-GAC AAA GAA ATG AAA GAT GT-3′	1728	^8,28^
	5′-GCA AAG ACT CTT GTC CCT GC-3′		
*S. mutans* serotype			
*c*	5′-CGGAGTGCTTTTTACAAGTGCTGG-3′	727	^28^
	5′-AACCACGGCCAGCAAACCCTTTAT-3′		
*e*	5′-CCTGCTTTTCAAGTACCTTTCGCC-3′	517	^28^
	5′-CTGCTTGCCAAGCCCTACTAGAAA-3′		
*f*	5′-CCCACAATTGGCTTCAAGAGGAGA-3′	316	^28^
	5′-TGCGAAACCATAAGCATAGCGAGG-3′		
*k*	5′-ATTCCCGCCGTTGGACCATTCC-3′	294	^28^
	5′-CCAATGTGATTCATCCCATCAC-3′		

### Serotyping of *cnm-*positive* S. mutan*s

Serotyping of *cnm*-positive *S. mutans* was performed by PCR using TaKaRa Ex Taq polymerase (Takara Bio) with serotype-specific primers for types *c*, *e*, and *f* (type *c*: forward, 5′-CGG AGT GCT TTT TAC AAG TGC TGG-3′ and reverse, 5′-AAC CAC GGC CAG CAA ACC CTT TAT-3′; type *e*: forward, 5′-CCT GCT TTT CAA GTA CCT TTC GCC-3′ and reverse, 5′-CTG CTT GCC AAG CCC TAC TAG AAA-3′; type *f*: forward, 5′-CCC ACA ATT GGC TTC AAG AGG AGA-3′ and reverse, 5′-TGC GAA ACC ATA AGC ATA GCG AGG-3′)^28^. Serotyping for type k was conducted using AmpliTaq Gold (Thermo Fisher Scientific, Waltham, MA, USA) with the following serotype-specific primers (forward, 5′-ATT CCC GCC GTT GGA CCA TTC C-3′; reverse, 5′-CCA ATG TGA TTC ATC CCA TCA C-3′)^28^. Template DNA and 1.5 mM MgCl₂ were included in all reactions, following the manufacturer’s protocols. A list of primers used in this study is provided in Table 3. PCR amplification conditions were as follows. For types *c*, *e*, and *f*: initial denaturation at 96 °C for 2 min, followed by 25 cycles of 96 °C for 15 s, 61 °C for 30 s, and 72 °C for 1 min, with a final extension at 72 °C for 5 min. For type *k*: initial denaturation at 94 °C for 5 min, followed by 30 cycles of 94 °C for 30 s, 60 °C for 30 s, and 72 °C for 30 s, with a final extension at 72 °C for 7 min. PCR products were subjected to electrophoresis on 1.5% or 0.7% agarose gels in Tris–acetate-EDTA buffer. Gels were stained with 0.5 μg/ml ethidium bromide and visualized under ultraviolet illumination.

### Clinical examination

All parents were asked to complete a questionnaire regarding the presence of systemic diseases. The oral status of each child was examined using a dental mirror and explorer under an operating light. The DMFT/dmft index for each child was calculated according to the criteria established by the World Health Organization^30^. Because some children were in the mixed dentition phase, dmft was used for primary teeth and DMFT for permanent teeth, and the combined total was evaluated.

### Collagen-binding assay

The collagen-binding properties of *S. mutans* strains were evaluated using previously described methods, with minor modifications^8^. A 10 mg/ml solution of type I collagen (Sigma-Aldrich Co.) prepared in 0.25 M acetic acid was used to coat 96-well tissue culture plates (Becton Dickinson, Franklin Lakes, NJ, USA), which were then incubated overnight at 4 °C. After incubation, the plates were washed three times with phosphate-buffered saline (PBS) and blocked with bovine serum albumin (Sigma-Aldrich Co.) in PBS for 1.5 h at 37 °C. Cultured bacterial cells were collected by centrifugation, washed, and diluted in PBS. A 100-μl aliquot of the bacterial suspension (1 × 10^9^ colony-forming units per well) was added to each well. Following 3 h of incubation at 37 °C, adherent cells were washed three times with PBS and fixed with 100 μl of 25% formaldehyde at room temperature for 30 min. After an additional three washes with PBS, the adherent cells were stained with 100 μl of 0.05% crystal violet (Fujifilm Wako Pure Chemical Industries) in water for 1 min. The wells were washed three times with PBS, and the bound dye was solubilized by the addition of 100 μl of 7% acetic acid. Absorbance was measured at 595 nm (OD_595_), and results were expressed as OD_595_ values after subtraction of readings from bovine serum albumin-coated control wells. The collagen-binding activity of each strain was expressed as a percentage relative to that of the positive control strain SA83 (Cnm +), which was defined as 100%. Data represent the mean ± standard deviation from four independent experiments conducted for each strain.

### Electron microscopy observations

Electron microscopy was conducted according to previously described methods^31,32^. For SEM, each bacterial sample was washed, fixed with 2% osmium tetroxide and 1% glutaraldehyde, dehydrated through a graded ethanol series, and dried using *t*-butyl alcohol via the freeze-drying method. The dried samples were mounted on specimen stages, coated with osmium for conductive treatment, and examined by SEM.

### Statistical analysis

The dmft/DMFT results are presented as the mean ± standard error. Statistical analyses were conducted using GraphPad Prism 9 (GraphPad Software Inc., La Jolla, CA, USA). Comparisons between two groups were performed using the Student’s *t*-test, and linear regression analysis was used to evaluate correlations with dmft/DMFT. *P *values < 0.05 were considered statistically significant.

## Data Availability

The datasets used and/or analyzed during the current study available from the corresponding author on reasonable request.
